# Strategies for Managing Arrhythmias in Patients with Cardiac Sarcoidosis

**DOI:** 10.19102/icrm.2019.100105

**Published:** 2019-01-15

**Authors:** Kent R. Nilsson, Venkateshwar Polsani, Thomas F. Deering

**Affiliations:** ^1^Department of Medicine, Augusta University/University of Georgia Medical Partnership, Athens, GA, USA; ^2^Piedmont Heart Institute, Piedmont Hospital System, Atlanta, GA, USA

**Keywords:** Arrhythmia, heart block, sarcoidosis, sudden death

## Abstract

Sarcoidosis is a systemic granulomatous disease that frequently involves the myocardium. Unfortunately, the sentinel manifestations of cardiac sarcoidosis are often potentially fatal bradyarrhythmia and tachyarrhythmia. Advanced imaging modalities such as cardiac magnetic resonance have allowed for increased diagnosis of cardiac involvement. The current review article explores diagnosis and treatment strategies for arrhythmias in patients with cardiac sarcoidosis.

## Introduction

Sarcoidosis is a disease of unknown etiology characterized pathologically by noncaseating granulomas.^1,2^ Although typically involving the lungs, sarcoidosis is a systemic disease that also frequently includes other organs in its scope such as the heart, liver, nervous system, and skin.^1^ Sarcoidosis occurs worldwide, but the highest rates are found in African-Americans and northern Europeans. In the United States, the annual incidence is estimated to be 10.9 per 100,000 in Caucasians and 35.5 per 100,000 in African-Americans, with women more likely to be afflicted than men.^3,4^ Autopsy and imaging studies of patients with pulmonary sarcoidosis suggest that more than 25% of patients have cardiac involvement, with only 5% exhibiting cardiac dysfunction.^3,5,6^ Manifestations of cardiac sarcoidosis include conduction abnormalities, ventricular arrhythmias, and heart failure, but presentation depends upon disease location and activity. Multiple studies have identified cardiac function as the strongest predictor of survival, with asymptomatic patients with normal ejection fractions (EFs) demonstrating an 89% to 100% 10-year survival rate, which can be contrasted against a 19% to 27% 10-year survival rate in patients with depressed EFs.^7,8^

## Diagnosis

Given the protean manifestations of sarcoidosis, diagnosis of cardiac sarcoidosis can be challenging. In 2014, the Heart Rhythm Society (HRS) attempted to address the lack of international guidelines by publishing expert consensus recommendations **(Table 1)**.^3^

**Figure 1:**
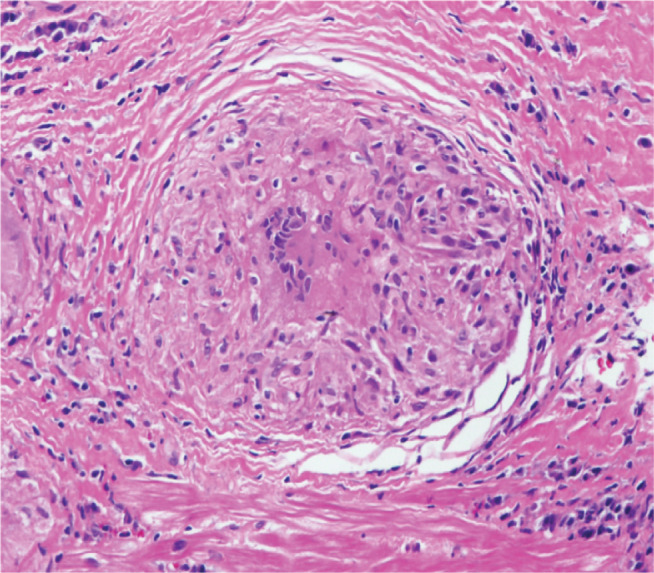
Noncaseating granuloma obtained from an endomyocardial biopsy of a patient with sarcoidosis, thereby confirming cardiac involvement.

Based upon these recommendations, the diagnosis of cardiac sarcoidosis can be accomplished either histologically, by a myocardial biopsy demonstrating noncaseating granulomas **(Figure 1)**, or clinically. The clinical diagnosis of cardiac sarcoidosis is much more nuanced and begins with a histological diagnosis of extracardiac sarcoidosis and the exclusion of other pathophysiologic explanations for the cardiac pathology. Under these circumstances, the clinical presentation of an unexplained cardiomyopathy, ventricular arrhythmia, or conduction block confirms the diagnosis of cardiac sarcoidosis. In the absence of clinical manifestations, ^18^F-fluorodeoxyglucose positron-emission tomography (FDG-PET) imaging, late gadolinium enhancement (LGE) on cardiac magnetic resonance (CMR) scan, or positive gallium uptake can confirm the diagnosis.

While histological diagnosis from an endomyocardial biopsy is the gold standard, overall sensitivity is low due to the patchy nature of disease involvement. As such, the committee recommended that, if endomyocardial biopsy is pursued, then it should be guided by either advanced imaging or electroanatomical mapping.^3,9,10^ In addition to advocating for imaging-guided biopsies, the committee recommended that screening be performed in patients with biopsy-proven extracardiac sarcoidosis for evidence of cardiac involvement **(Table 2)**. Specifically, they suggested a class I recommendation for asking patients about unexplained syncope, presyncope, and palpitations and for screening electrocardiography and a class IIa recommendation for screening echocardiography, respectively. Advanced cardiac imaging (eg, CMR imaging) is recommended if any result of the aforementioned screening suggests cardiac involvement.

### Cardiac magnetic resonance imaging

**Figure 2:**
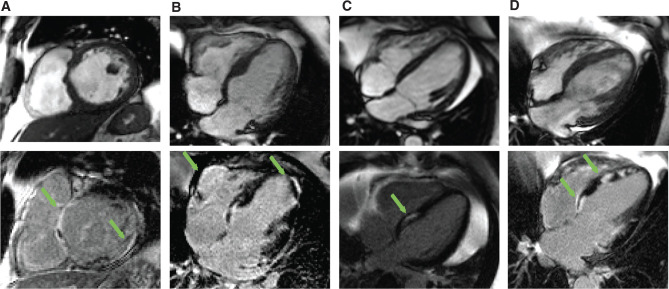
CMR scans of four patients with sarcoidosis and evidence of cardiac involvement. Both noncontrast (top row) and LGE (bottom row) images are shown. **A:** Patient 1 demonstrates evidence of myocardial fibrosis with delayed enhancement of the interventricular septum and inferior and lateral walls (arrows). **B:** Patient 2 demonstrates a noncoronary-artery-disease pattern of delayed hyperenhancement with involvement of the right ventricular aneurysm, basal inferior septum, and lateral wall of the left ventricle. **C:** Patient 3 demonstrates evidence of focal midmyocardial scar in the region of the His-Purkinje system and the basal inferior septum. **D:** Finally, patient 4 has evidence of diffuse involvement.

CMR imaging demonstrating abnormal LGE strongly supports the diagnosis of cardiac sarcoidosis.^11–14^ Although there is no single pattern of delayed enhancement that is pathognomonic for cardiac involvement, patchy regions of LGE localized in the basal and subepicardial regions are often seen **(Figure 2)**.^15,16^

### Cardiac ^18^F-fluorodeoxyglucose positron-emission tomography imaging

**Figure 3:**
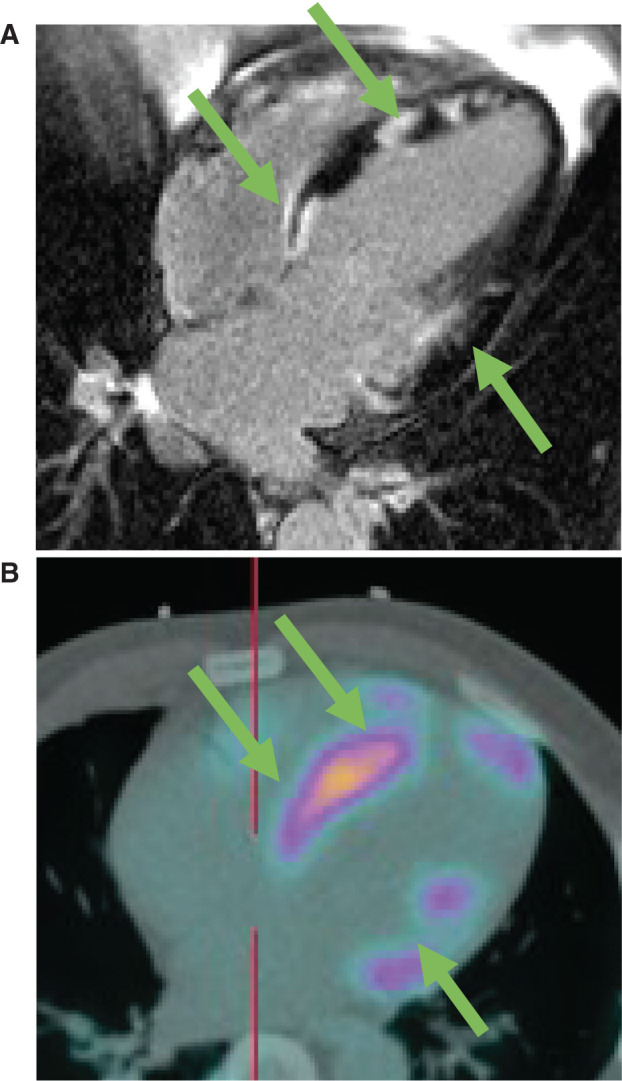
**A:** Delayed hyperenhancement of a patient with cardiac sarcoidosis demonstrates diffuse, patchy involvement in areas throughout the right and left ventricles (arrows). **B:** FDG-PET demonstrates active inflammation in areas that correspond to areas of LGE. Courtesy of Steven R. Sigman, MD.

Owing to its unique ability to image activated macrophages and inflammation, FDG-PET imaging has proven to be a useful imaging modality in diagnosing a patient, determining disease activity, and monitoring the response to therapy.^17–19^ Of the three different patterns of uptake seen in patients with cardiac sarcoidosis—diffuse, focal, and focal on diffuse—focal FDG uptake is the most common **(Figure 3)**. As in the case with CMR, image acquisition and interpretation need to performed at a center with appropriate expertise.^3^ Pooled estimates from a recent meta-analysis of seven studies including 164 patients demonstrated that FDG-PET had a sensitivity of 89% and a specificity of 78% for diagnosing cardiac sarcoidosis.^20^

As FDG-PET and CMR detect different pathologic features of cardiac involvement, combining the two imaging modalities could increase both sensitivity and specificity. To that end, Vita et al.^21^ recently published a retrospective analysis of 107 consecutive patients who underwent both CMR and FDG-PET imaging. They found that a significant percentage of patients with evidence of cardiac sarcoidosis on FDG-PET did not have evidence of the same on CMR and vice versa. Specifically, only 60 (66%) of the 91 patients with LGE had abnormal FDG uptake. When FDG-PET was combined with CMR imaging, close to half (48/107) had the likelihood of their disease reclassified. Given these findings, multimodality imaging likely offers the most comprehensive approach to diagnosis, prognosis, and treatment.

## Bradyarrhythmias and conduction system abnormalities

Conduction system abnormalities are a common and often sentinel, clinical manifestation of cardiac sarcoidosis due to granulomatous involvement in the basal interventricular septum **(Figure 2)**. Estimated to affect up to 30% of patients with biopsy-proven cardiac sarcoidosis, disruption of the conduction system is more common during the active phases of the disease.^22^ In addition to affecting patients with known cardiac sarcoidosis, heart block can be an initial manifestation of sarcoidosis. The case series of Kandolin et al.^23^ of adults aged 18 years to 55 years demonstrated that 19% of patients younger than 55 years with idiopathic complete heart block had cardiac sarcoidosis. Similarly, Nery et al.^24^ found that 34% (11/32) of middle-aged patients (aged 18–60 years) with idiopathic complete heart block had cardiac sarcoidosis.

In addition to its inherent morbidity and mortality, infra-Hisian conduction disease is often a harbinger of ventricular arrhythmias, which raises questions about the implantation of a pacemaker versus an implantable cardioverter-defibrillator (ICD), even when the disease can be effectively suppressed with corticosteroids.^25^ To our knowledge, six studies have sought to evaluate the efficacy of steroids in the treatment of cardiac sarcoidosis arising from complete heart block. In total, the studies included 73 patients (57 receiving steroids, 16 not receiving steroids). No patient in the control arms had atrioventricular node recovery. In contrast, 47.4% (27/57) in the corticosteroid arms did.^26^ Collectively, these studies suggest that immunosuppression with steroids should be considered in all patients with advanced infra-Hisian conduction disease and sarcoidosis. Although there is no consensus regarding treatment doses available at this time, an initially intense dose of prednisone (0.5 mg/kg/day) tapered overtime to a lower maintenance dose (5–10 mg/day) is reasonable.^27^

Given the variable disease course and response to therapy, it is our practice for all patients with advanced infra-Hisian conduction disease to receive a permanent pacing system before beginning immunosuppression. Moreover, given the potential for fatal ventricular arrhythmias, we routinely implant dual-chamber ICDs (or cardiac resynchronization therapy defibrillators where appropriate), consistent with the class IIa recommendation of the expert consensus panel.^3^

## Atrial arrhythmias

Although atrial involvement in cardiac sarcoidosis is common pathologically, its role in atrial dysrhythmias remains a source of debate. A recent study examining patients who meet the HRS’ expert panel criteria for cardiac sarcoidosis found that 32% of patients had supraventricular arrhythmias, of which atrial fibrillation (AF) was the most prevalent.^28^ This, however, is similar to the lifetime risk of AF in otherwise-healthy patients in the Framingham study.^29^ Pathophysiologically, it is reasonable to speculate that granulomatous infiltration coupled with increased atrial pressure arising from both the diastolic and systolic sequela of ventricular involvement would increase the risk of AF.

Although patients with sarcoidosis are at increased risk of venous thromboembolism, suggesting the existence of a hypercoagulable state, there are no formal recommendations with regard to stroke prophylaxis outside of the published guidelines for thromboprophylaxis in nonvalvular AF.^30^ It is our practice, however, to incorporate this information in shared decision-making efforts when discussing anticoagulation.

To date, there are little data available regarding the efficacy of either antiarrhythmic drugs or ablation in the treatment and management of arrhythmias arising from cardiac sarcoidosis.^3^ The pathology of cardiac sarcoidosis is defined by granulomas, fibrosis, and scarring. As such, the attendant alterations in conduction velocity and tissue refractoriness make the use of class Ic antiarrhythmic drugs potentially proarrhythmic.^31^ Accordingly, the HRS consensus statement notes that “antiarrhythmic therapy with class I agents is not recommended,” giving it a class III recommendation.^3^

The utility of pulmonary vein isolation for the treatment of atrial dysrhythmias is similarly of unclear benefit. A case series of nine patients with AF and cardiac sarcoidosis found that, through 1.8 years ± 1.9 years of follow-up, the rate of success was comparable to that in the general population.^32^ Given the limited number of patients included in this study, coupled with the heterogeneity of the ablation lesion sets created by operators, it is not possible to generalize the results of this study. To that end, Srivatsa and Rogers^33^ reported on a patient with cardiac sarcoidosis and AF who derived no benefit from pulmonary vein isolation but who showed effective arrhythmia suppression with immunosuppression.

## Ventricular arrhythmias

Ventricular arrhythmias are a common manifestation of cardiac sarcoidosis and are more likely to occur in patients with infra-Hisian conduction disease and/or depressed EF.^34^ Although triggered activity and enhanced automaticity can result in frequent ventricular ectopy that is responsive to immunosuppression,^35,36^ the majority of ventricular arrhythmias are believed to be reentrant arrhythmias around scar tissue.^37,38^ Treatment involves a combination of immunosuppression, antiarrhythmic drug therapy, and radiofrequency ablation.

### Immunosuppression

Data on the efficacy of immunosuppression in treating ventricular tachycardia (VT) are scant and at times contradictory, likely representing a heterogeneous collection of patients and disease states.^34,39^ The study of Yodogawa et al.^36^ of 31 patients with cardiac sarcoidosis and ventricular arrhythmias demonstrated that patients with less severe disease, as measured by EF, responded better to steroids than did those with more severely depressed EFs. As steroids reduce inflammation, these results are in agreement with a management strategy of using aggressive immunosuppression early in the disease process to prevent fibrosis. Unfortunately, patients with more advanced disease may see an increase in arrhythmias following the initiation of steroids. The study of Segawa et al.^34^ of 68 patients with cardiac sarcoidosis who were started on corticosteroids demonstrated that 20 patients (29%) went on to develop ventricular arrhythmias after the initiation of steroids. It is unclear as to whether the emergence of ventricular arrhythmias after the initiation of steroids was due to immunomodulation or more aggressive disease.^34^

### Antiarrhythmic drug therapy

Although antiarrhythmic drugs are commonly used to treat ventricular arrhythmias, data on their use are still quite limited. In general, class III agents are preferred (eg, amiodarone or sotalol), as class Ic drugs are contraindicated in the setting of structural heart disease.^39^ In patients with electrical storm, it is recommended that patients receive both immunosuppression and amiodarone.^3^

### Ablation of ventricular arrhythmias

For roughly half of patients with VT, medical therapy fails to suppress their irregular rhythms.^2,40^ In such cases, radiofrequency ablation is indicated. Unlike ischemic VTs, which are predominantly found in the left ventricle, the heterogeneous, patchy fibrosis that predisposes cardiac sarcoid patients to ventricular arrhythmias can be found in the right ventricle, left ventricle, and epicardium.^11,38^ Preprocedure imaging is, therefore, crucial to the successful identification and ablation of critical isthmuses. To the best of our knowledge, five studies of a total of 83 patients with cardiac sarcoidosis undergoing catheter ablation for VT have been published to date. One hundred percent of these patients endocardial ablation, whereas 18% also underwent epicardial ablation. Furthermore, 88.4% of these patients either were arrhythmia-free postoperation or experienced a significant reduction in overall VT burden.^41^

## Sudden cardiac death prevention

As discussed above, patients with cardiac sarcoidosis have an increased incidence of potentially fatal ventricular arrhythmias. For those with an EF of 35% or less despite using guidelines-directed medical therapy or who have experienced hemodynamically significant ventricular arrhythmias, traditional primary and secondary indications for device implantation apply.^39,42,43^ In addition, it is widely accepted that patients with advanced conduction disease requiring permanent pacing would likely derive benefit from a primary prevention ICD. As evidence of the particularly malignant nature of sarcoidosis-induced ventricular arrhythmias, the annual incidence of appropriate shocks for primary prevention ICDs in patients with cardiac sarcoidosis is roughly threefold higher (10%–15% per year) than what is reported in primary prevention trials.^44,45^ More importantly, patients with EFs of more than 35% are still at risk for malignant arrhythmias. For patients with cardiac sarcoidosis and an EF of between 36% and 49%, additional insight into their risk of sudden cardiac death can be obtained with CMR and FDG-PET imaging and electrophysiology study.

### Cardiac magnetic resonance imaging for risk stratification

As mentioned above, CMR serves an invaluable role in both diagnosing cardiac sarcoidosis and guiding therapy for it **(Figure 2)**. Its ability to predict the risk of sudden cardiac death has further proven useful in patients with mild to moderate reductions in EF. In a study of 155 patients with cardiac sarcoidosis, Greulich et al.^13^ demonstrated that the 25.5% of patients with LGE had significantly higher risks of VT, aborted sudden cardiac death, ICD discharge, and death than did patients without LGE. Additionally, whereas 28.2% of patients with LGE experienced the primary endpoint during a median follow-up of 2.6 years, only 0.9% of LGE-negative patients did. The Cox hazard ratio of 31.6 for LGE was superior to that of left ventricular EF. Moreover, except for one patient dying from a noncardiac cause, no patient without LGE died or experienced any event during follow-up, even in the presence of a severely reduced EF. Building on Greulich et al.’s study, a recent meta-analysis of 760 patients from 10 studies demonstrated that the presence of LGE was associated with a 10-fold higher rate of ventricular arrhythmias, ICD discharges, or sudden cardiac death (odds ratio: 10.74; p < 0.00001).^14^ As such, CMR is recommended as part of a risk stratification protocol in at-risk patients who do not meet traditional primary or secondary indications.

### ^18^F-fluorodeoxyglucose positron-emission tomography for risk stratification

Similar to CMR, the role of FDG-PET in risk stratification is emerging **(Figure 3)**. A recent study by Blankstein et al.^17^ compared abnormal versus normal FDG-PET imaging in 118 patients with cardiac sarcoidosis. Patients with abnormal FDG perfusion (n = 71) were significantly more likely to experience VT over the 1.5 median years of follow-up. The negative predictive value, however, was less than that seen with CMR. As such, the HRS expert consensus statement favors CMR over FDG-PET for risk stratification, stating that there were “insufficient data” to include a recommendation on FDG-PET for use in sudden cardiac death risk stratification.^3^

### Electrophysiology study for risk stratification

In patients with biopsy-proven extracardiac sarcoidosis and imaging results that are suggestive of cardiac involvement (according to either CMR or FDG-PET imaging), electrophysiologic testing can provide additional risk stratification. In a study of 76 patients with cardiac sarcoidosis, 75% of patients with a positive electrophysiology study versus 1.5% of those who were not inducible experienced a ventricular arrhythmia or death.^46^ It is important to note that it is unclear whether inducibility is more predictive than left ventricular EF alone, as inducible patients had significantly lower EFs than did noninducible patients (36.4% ± 4.2% versus 55.8% ± 1.5%).

### Summary of risk stratification for patients with an ejection fraction of 36% to 49%

**Figure 4:**
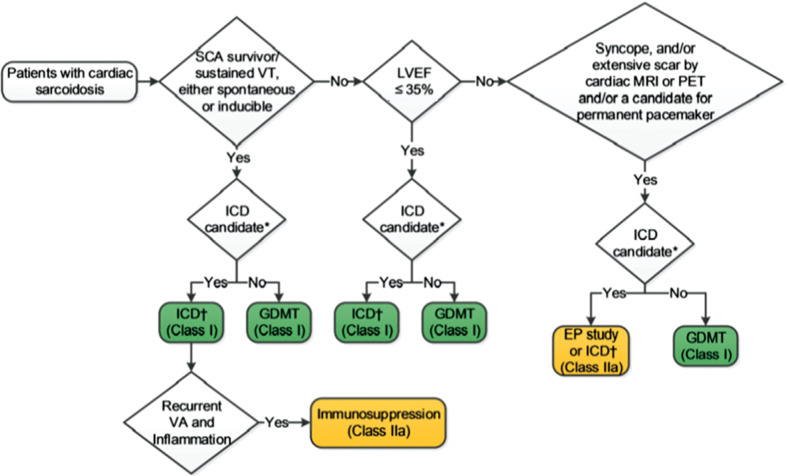
2017 AHA/ACC/HRS recommendations for the prevention of sudden cardiac death in patients with cardiac sarcoidosis.^39^ *ICD candidacy as determined by functional status, life expectancy, or patient preference. †For recurrent sustained monomorphic VT, refer to **Figure 2**. Reproduced with permission from: Al-Khatib SM, Stevenson WG, Ackerman MJ, et al. 2017 AHA/ACC/HRS guideline for management of patients with ventricular arrhythmias and the prevention of sudden cardiac death: a report of the American College of Cardiology/American Heart Association Task Force on Clinical Practice Guidelines and the Heart Rhythm Society. *J Am Coll Cardiol.* 2017 Oct 25. pii: S0735-1097(17)41306-4. [Epub ahead of print]. EP: electrophysiology; GDMT: guidelines-directed management and therapy; ICD: implantable cardioverter-defibrillator; LVEF: left ventricular ejection fraction; MRI: magnetic resonance imaging; PET: positron-emission tomography; SCA: sudden cardiac arrest; SCD: sudden cardiac death; VA: ventricular arrhythmia; VT: ventricular tachycardia.

Patients with cardiac involvement who have a reduced EF not meeting primary prevention criteria are clearly at increased risk of sudden cardiac death. Based upon EF alone, the HRS consensus committee gave a class IIb recommendation of “might be considered” to the implantation of an ICD. For patients with an EF of 36% to 49%, a CMR scan with LGE, and inducible ventricular arrhythmias on electrophysiology study, this was upgraded to a class IIa recommendation.^3^ The more recent 2017 American Heart Association/American College of Cardiology/HRS (AHA/ACC/HRS) consensus guidelines on VT include similar recommendations, with the exception of that, for patients with cardiac sarcoidosis and an EF of 35% or more, a positive electrophysiology study for ventricular tachycardia is sufficient to justify an ICD **(Figure 4)**.^39^ The benefits of ICD implantation, however, need to be balanced against the risk of inappropriate ICD therapies, which are reported to be as high as 11.6%.^47^

## Conclusion

Cardiac sarcoidosis is a rare condition with potentially fatal complications. Advanced imaging has allowed for improved diagnosis, treatment, and risk stratification. Management of both bradyarrhythmia and tachyarrhythmia begins with immunosuppression. For patients not meeting traditional primary or secondary indications for ICDs, additional forms of risk stratification including electrophysiology study and CMR and FDG-PET imaging are indicated. Given the low overall prevalence of cardiac sarcoidosis, large prospective registries are needed to define disease progression, optimize treatment, and optimally risk-stratify patients.

## Figures and Tables

**Table 1:** Criteria for the Diagnosis of Cardiac Sarcoidosis^3^

Pathway to Diagnosis No.	Details
1	• Histological diagnosis from myocardial biopsy demonstrating noncaseating granulomas
2	• Clinical diagnosis (histological diagnosis of extracardiac sarcoidosis) • One or more of the following: ○ Immunosuppressant responsive cardiomyopathy or heart block ○ Unexplained reduced LVEF (< 40%) ○ Unexplained sustained VT ○ Advanced infra-Hisian conduction disease (eg, Mobitz type II second degree) ○ Cardiac FDG-PET imaging consistent with cardiac sarcoidosis ○ LGE on CMR consistent with sarcoidosis and/or positive gallium uptake • Other causes have been excluded

LVEF: left ventricular ejection fraction; VT: ventricular tachycardia; FDG-PET: ^18^F-fluorodeoxyglucose positron-emission tomography; LGE: late gadolinium enhancement; CMR: cardiac magnetic resonance.

**Table 2:** Recommendations for Screening for Cardiac Involvement in Patients with Biopsy-proven Extracardiac Sarcoidosis^3^

Class	Recommendation
Class I	• All patients with biopsy-proven extracardiac sarcoidosis should be asked about unexplained syncope/presyncope/significant palpitations and have a 12-lead ECG recorded to evaluate for infra-Hisian conduction disease
Class II	• Screening for cardiac involvement with an echocardiogram (eg, depressed ejection fraction, regional wall motion abnormalities) can be useful in patients with extracardiac sarcoidosis • Advanced cardiac imaging (ie, CMR or FDG-PET) at an experienced center can be useful in patients with one or more screening abnormalities (eg, ECG conduction abnormalities)
Class III	• Advanced cardiac imaging (CMR or FDG-PET) is not recommended in patients without abnormalities on initial screening

ECG: electrocardiogram; FDG-PET: ^18^F-fluorodeoxyglucose positron-emission tomography; CMR: cardiac magnetic resonance.
